# Intranasal Lipid Nanoparticles Containing Bioactive Compounds Obtained from Marine Sources to Manage Neurodegenerative Diseases

**DOI:** 10.3390/ph16020311

**Published:** 2023-02-16

**Authors:** Joana Torres, Inês Costa, Andreia F. Peixoto, Renata Silva, José Manuel Sousa Lobo, Ana Catarina Silva

**Affiliations:** 1 UCIBIO, REQUIMTE, Laboratory of Pharmaceutical Technology/Centre of Research in Pharmaceutical Sciences, Faculty of Pharmacy, University of Porto, 4050-313 Porto, Portugal; 2 Associate Laboratory i4HB-Institute for Health and Bioeconomy, Faculty of Pharmacy, University of Porto, 4050-313 Porto, Portugal; 3 UCIBIO, REQUIMTE, Laboratory of Toxicology, Department of Biological Sciences, Faculty of Pharmacy, University of Porto, 4050-3131 Porto, Portugal; 4 LAQV/REQUIMTE, Department of Chemistry and Biochemistry, Faculty of Sciences, University of Porto, 4169-007 Porto, Portugal; 5 FP-I3ID (Instituto de Investigação, Inovação e Desenvolvimento), FP-BHS (Biomedical and Health Sciences Research Unit), Faculty of Health Sciences, University Fernando Pessoa, 4200-150 Porto, Portugal

**Keywords:** antioxidants, marine bio-waste, bioactive compounds, neurodegenerative diseases, nanostructured lipid carriers, NLC, solid lipid nanoparticles, SLN, intranasal administration, nose-to-brain

## Abstract

Marine sources contain several bioactive compounds with high therapeutic potential, such as remarkable antioxidant activity that can reduce oxidative stress related to the pathogenesis of neurodegenerative diseases. Indeed, there has been a growing interest in these natural sources, especially those resulting from the processing of marine organisms (i.e., marine bio-waste), to obtain natural antioxidants as an alternative to synthetic antioxidants in a sustainable approach to promote circularity by recovering and creating value from these bio-wastes. However, despite their expected potential to prevent, delay, or treat neurodegenerative diseases, antioxidant compounds may have difficulty reaching the brain due to the need to cross the blood–brain barrier (BBB). In this regard, alternative delivery systems administered by different routes have been proposed, including intranasal administration of lipid nanoparticles, such as solid lipid nanoparticles (SLN) and nanostructured lipid carriers (NLC), which have shown promising results. Intranasal administration shows several advantages, including the fact that molecules do not need to cross the BBB to reach the central nervous system (CNS), as they can be transported directly from the nasal cavity to the brain (i.e., nose-to-brain transport). The benefits of using SLN and NLC for intranasal delivery of natural bioactive compounds for the treatment of neurodegenerative diseases have shown relevant outcomes through in vitro and in vivo studies. Noteworthy, for bioactive compounds obtained from marine bio-waste, few studies have been reported, showing the open potential of this research area. This review updates the state of the art of using SLN and NLC to transport bioactive compounds from different sources, in particular, those obtained from marine bio-waste, and their potential application in the treatment of neurodegenerative diseases.

## 1. Introduction

In recent years, the average consumption of fish, shellfish, and crustaceans has increased significantly, as they can contribute positively to human health and well-being, when combined with a healthy lifestyle [1,2]. However, this increase in the consumption of marine organisms has led to the annual production of tens of millions of tons of solid waste resulting from their processing. Currently, the Food and Agriculture Organization of the United Nations (FAO) recognizes the environmental, social, and economic problems resulting from the landfilling of this waste [1,3,4]. To overcome this challenge, an innovative solution has been proposed, consisting of the recovery and valorization of waste resulting from the processing of marine organisms, as this bio-waste is a rich reservoir of various bio-functional components [2,5]. There are already many investigations that demonstrate the potential of using these products to obtain bioactive compounds with different activities (e.g., anticancer, antimicrobial, antioxidant, and immunomodulatory) that can be used to develop value-added products in the pharmaceutical industry for the treatment of different diseases [2,3,4,6,7]. For example, bioactive compounds that can be isolated from shrimp waste include the chito-oligosaccharides present in chitin or chitosan, omega-3, and astaxanthin. Salmon nasal cartilage is a valuable source of proteoglycans with anti-angiogenic activity. Fish skin is an important source of collagen, which can be hydrolyzed to bioactive peptides. Algae contain high amounts of phytonutrients, particularly those belonging to the gender *Chlorophyta*, *Rhodophyta*, and *Phaeophyta*, which are rich in dietary fibers, omega-3, β-carotene, astaxanthin, vitamin C, and other compounds beneficial to human health [3,8].

The scientific community already recognizes the extraordinary potential of bioactive compounds obtained from marine bio-waste to prevent and treat various diseases, such as those showing antioxidant activity that can prevent, delay, or treat neurodegenerative diseases. Indeed, within the circular economy paradigm, the use of this bio-waste has multiple benefits, promoting a more sustainable aquaculture and fishing industries, and reducing the impact of anthropic exploitation of marine resources [1,9,10,11]. However, despite the potential of these new bioactive compounds, there is still no effective therapeutic solution for neurodegenerative diseases. Researchers have been pointed that the main challenge is the difficulty for molecules to cross the blood–brain barrier (BBB) to reach the brain. Different approaches have been investigated to circumvent this problem. Among them, the use of lipid nanoparticles (i.e., solid lipid nanoparticles—SLN and nanostructured lipid carriers—NLC), administered by alternative routes, such as the intranasal (i.e., nose-to-brain route), has been described as the most promising option [12,13,14,15,16,17].

This review work begins with a description of the different pathophysiological mechanisms underlying neurodegenerative diseases, followed by the presentation of examples of bioactive compounds obtained from marine bio-waste with potential antioxidant activity in the management of these diseases. Finally, the state-of-the-art use of intranasal SLN and NLC to transport bioactive compounds directly to the brain, promoting the treatment of neurodegenerative diseases, is presented.

## 2. Neurodegenerative Diseases

Neurodegenerative diseases are a group of debilitating conditions that result from progressive damage inflicted on cells and nervous system, with abnormal deposition of proteins and the progressive loss of synapses and neurons [18,19]. Due to the different pathophysiological mechanisms underlying these diseases, they present a wide spectrum of clinical manifestations. With neurodegenerative disease progression, the severity of the symptoms gradually increases, resulting in a reduced ability to live independently and in a huge impact on the patients’ quality of life [19].

Some examples of neurodegenerative diseases include Alzheimer’s disease, vascular dementia, frontotemporal dementia, mixed dementia, and dementia with Lewy bodies, which are characterized by cognitive deficits and memory loss. On the other side, neurodegenerative diseases that mainly affect the locomotor system include Amyotrophic lateral sclerosis, Huntington’s disease, Parkinson’s disease, Multiple sclerosis, and Spinocerebellar ataxias [20,21]. In the present review, the most prevalent and debilitating neurodegenerative diseases will be explored, namely Alzheimer’s disease, Parkinson’s disease, multiple sclerosis, and amyotrophic lateral sclerosis.

Alzheimer’s disease is the most common neurodegenerative disease, corresponding to 60% to 80% of cases of dementia [22]. This illness was described for the first time in 1906 by Alois Alzheimer, and is characterized by the extracellular deposition of amyloid-β (Aβ) peptide in senile plaques, by the intraneuronal accumulation of hyperphosphorylated tau protein (leading to the formation of intracellular neurofibrillary tangles), as well as by oxidative stress, neuroinflammation, ferroptosis, and synaptic loss [23,24]. The main symptoms expressed by the patients are persistent and frequent memory difficulties, vague speech, delay in performing routine activities, emotional unpredictability, and inability to understand questions and instructions [25].

Parkinson’s disease is a complex neurological disease with early death of dopaminergic neurons in *substantia nigra pars compacta* and is characterized by Lewy bodies formation, oxidative stress, iron overload, mitochondrial dysfunction, ferroptosis, and neuroinflammation. It affects about 0.1–0.2% of the population, and patients experience motor symptoms such as tremor, bradykinesia, rigidity, and postural instability [26,27], and also non-motor symptoms such as depression and sleep problems [28].

Multiple sclerosis is recognized as a chronic inflammatory and demyelinating disease that affects 2.1 million people worldwide [29]. The defects in oligodendrocyte regeneration and myelin damage leads to axonal degeneration, which constitutes the main cause for the progression of the irreversible neuronal destruction that leads to permanent disability [30]. Symptoms experienced by patients include walking impairment, weakness, cognitive impairment, depression, and fatigue [31].

Amyotrophic lateral sclerosis is a neurodegenerative disease characterized by the selective dysfunction and loss of motor neurons in specific brain regions, with aggregation and accumulation of ubiquitinated proteinaceous inclusions, consequently leading to paralysis and death [32,33,34]. This neurodegenerative disease has an incidence of approximately 1.2–6 per 100.000 persons annually [34]. In most cases of amyotrophic lateral sclerosis, there is no family history associated, but in about 10% of cases, a dominantly inherited autosomal mutation occurs in distinct genes, such as in superoxide dismutase 1 (SOD1), C9orf72, and TAR DNA-binding protein 43 (TDP-43) genes [35]. The main symptoms are progressive muscle weakness, slowness of movements with muscle stiffness, muscle atrophy, and muscle cramps [33]. In the next sub-section, the main pathophysiological mechanisms common to these neurodegenerative diseases will be addressed.

### 2.1. Main Pathophysiological Mechanism Underlying Neurodegenerative Diseases

**Figure 1:**
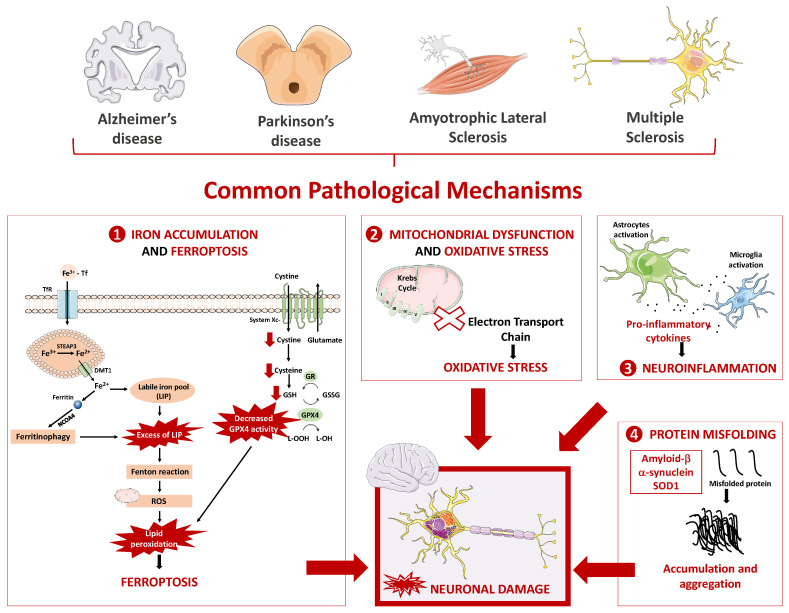
Common pathophysiological mechanisms underlying the most prevalent and debilitating neurodegenerative diseases. Neurodegenerative diseases are a group of debilitant conditions that result from the progressive damage inflicted to the neuronal cells and nervous system, with abnormal deposition of proteins, and with the progressive loss of synapses and neurons. Alzheimer’s disease, Parkinson’s disease, Amyotrophic Lateral Sclerosis, and Multiple Sclerosis are examples of complex neurodegenerative diseases sharing several common pathophysiological mechanisms, such as: (1) iron overload, (2) mitochondrial dysfunction and oxidative stress, (3) neuroinflammation, and (4) protein misfolding. Iron has essential functions in the brain and, therefore, needs to cross the blood–brain barrier (BBB) to reach this organ. The most elucidating hypothesis of the passage of iron through the luminal membrane of the capillary endothelium mainly occurs through the transferrin/transferrin receptor (Tf/TfR) pathway. This process starts with the binding of the complex ferric iron (Fe^3+^)-Tf to the extracellular portion of transferrin receptor (TfR), followed by the endocytosis of the complex, formation of endosome, and acidification of the microenvironment within endosome. Next, occurs the dissociation of iron from Tf and the reduction of ferric iron (Fe^3+^) to Fe^2+^ by the ferrireductase six-transmembrane epithelial antigen of prostate 3 (STEAP3). Fe^2+^ accumulates in cytoplasm, forming the labile iron pool (LIP), and the excess of intracellular iron is then stored in ferritin. Ferritinophagy is defined as the autophagic degradation of ferritin, a process mediated by nuclear receptor coactivator 4 (NCOA4). Ferritin, in combination with NCOA4, is transported to the lysosomes for degradation, being then the iron released for cellular physiological activities. However, when this metal is in excess, it participates in Fenton reaction leading to a cycle between the two redox states and prompting the generation of •OH, promoting lipid peroxidation and ferroptosis, a new type of regulated cell death. Ferroptosis is also characterized by an inhibition of System Xc-, with the consequent decrease in glutathione peroxidase 4 (GPX4) activity and promotion of lipid peroxidation, leading to neuronal damage. Mitochondria are essential organelles for eukaryotic life, producing most of the energy or adenosine triphosphate (ATP) required by the cell, being responsible for cellular respiration and oxidative phosphorylation. Changes in the correct functioning or in structures involved in this process lead to a decrease in ATP production, to the accumulation of reactive oxygen species (ROS), and to the release of apoptosis-inducing factors, leading to oxidative stress and cell death. Neuroinflammation is another pathological mechanism present in neurodegenerative diseases, and results from the presence of chronically activated glial cells (astrocytes and microglia) in the brain, which release cytokines and chemokines that are toxic to neurons. Finally, protein misfolding and aggregation of specific proteins into toxic products is a common feature of neurodegenerative diseases. Depending on the type of protein involved and the pathology in question, its aggregation promotes different consequences. For example, in Alzheimer’s disease, amyloid beta peptide (Aβ), originating from the fragmentation of amyloid precursor protein (APP), accumulates in the brain in the form of senile plaques. In Parkinson’s disease, α-synuclein (α-syn) is often found accumulated and aggregated and has several harmful effects. GR: Glutathione reductase; GSH: Reduced glutathione; GSSG: Glutathione disulfide.

Although the mentioned neurodegenerative diseases are complex and present different symptoms and underlying mechanisms, several common mechanisms have been studied aiming to explain the development and progression of these pathologies. Figure 1 summarizes the main pathophysiological mechanisms that appear to be common to distinct neurodegenerative diseases, including oxidative stress and mitochondrial dysfunction, neuroinflammation, protein misfolding, and iron overload and ferroptosis.

#### 2.1.1. Oxidative Stress and Mitochondrial Dysfunction

Oxidative stress is considered a state in which free radicals and their products are in excess when compared to the levels of antioxidant defenses. Under normal cellular conditions, reactive oxygen species (ROS) and reactive nitrogen species (RNS) play an important physiological role, and their intracellular concentrations are kept at low or moderate levels by an endogenous antioxidant system. When the production of ROS/RNS surpasses the capacity of the endogenous antioxidant system, the onset of several adverse mechanisms is observed, such as interaction with lipids, proteins, and DNA, which contribute to cell degeneration [36].

The brain has several features that make it very susceptible to oxidative stress [37]: (i) membrane lipids contain high levels of polyunsaturated fatty acids (PUFAs) that are the preferred substrate for lipid peroxidation; (ii) high consumption of oxygen that contributes to the generation of superoxide anions; (iii) lower concentrations of antioxidant enzymes (catalase—CAT, superoxide dismutase—SOD and glutathione peroxidase—GPx); (iv) high concentration of iron, which promotes participation in the Fenton reaction and in the generation of ROS.

Mitochondria are essential organelles for eukaryotic life, producing most of the energy or adenosine triphosphate (ATP) required by the cell, being responsible for cellular respiration and oxidative phosphorylation, and also being involved in maintaining calcium levels at physiological concentrations in the cytosol and intervening in the apoptotic cell death mechanism. The process of oxidative phosphorylation occurs via electron transport chain, consisting of four complexes that transfer electrons from NADH (nicotinamide adenine dinucleotide) and FADH2 (flavin adenine dinucleotide) to molecular oxygen. The energy released by the oxidation of these substrates is used to generate a proton gradient in the mitochondrial membrane that will be used in complex V for the synthesis of ATP. Changes in the correct functioning or structures of this process originates a decrease in ATP production, to the accumulation of ROS, and to the release of apoptosis-inducing factors, leading to cell death [38]. This organelle is the main generator of ROS, but also its main target. The process of oxidative phosphorylation involves the interaction between unpaired electrons with molecular oxygen (O_2_), leading to the generation of superoxide anion (O2^•−^). This radical is further converted in H_2_O_2_ by SOD. In the presence of Ferrous iron (Fe^2+^), H_2_O_2_ can be converted into the highly reactive hydroxyl radical though the Fenton reaction, leading to oxidative damage [39,40,41].

Mitochondria undergo constant morphological changes by the process of continuous cycles of fusion and fission. The balance between these two processes determines the function of this organelle, controls its bioenergetic function and mitochondrial turnover, and protects mitochondrial DNA [42,43]. Besides, as mentioned, mitochondria play a pivotal role in maintaining the normal Ca^2+^ homeostasis. This cation is transported across the inner mitochondrial membrane via the electrogenic mitochondrial calcium transporter [44]. Changes in the mitochondrial influx/efflux of Ca^2+^ leads to a deregulation of mitochondrial Ca^2+^ homeostasis and, consequently, in mitochondrial Ca^2+^ overload. This Ca^2+^ overload induces oxidative stress and the opening of permeability transition pore, which can be an initial trigger for apoptotic and necrotic cell death. Besides that, it can also stimulate the activity of nitric oxide synthetase to generate NO•, which results in inhibition of via electron transport chain and leads to subsequent ROS production [45].

The connection of Parkinson’s disease, mitochondrial dysfunction, and oxidative stress has been proven in many studies. For example, in 2016 a study concluded that Parkinson’s disease is associated with increased levels of oxidative biomarkers, such as lipid peroxides and malondialdehyde and SOD activity, and inversely correlated with the levels of antioxidant defenses, such as the total radical trapping antioxidant parameter, SH-groups, and catalase activity, promoting oxidative stress and cell damage [46]. In the case of amyotrophic lateral sclerosis, a relationship was found between disease progression and glutathione peroxidase 4 (GPX4) levels, an enzyme belonging to the antioxidant system, which is responsible for preventing the formation of lipid peroxides. A group observed that in a mouse model of ferroptosis with GPX4 neuronal inducible knockout, the ablation of GPX4 in neurons resulted in a rapid paralysis and severe muscle atrophy, which are features of amyotrophic lateral sclerosis [47].

For Alzheimer’s disease, Zweig and colleagues analyzed the protective effects of *Centella asiatica* (a natural compound with antioxidant properties) in five FAD mice. The group concluded that *Centella asiatica* improved spatial and contextual memory, with concomitant increased antioxidant gene expression and a decrease in the Aβ plaque burden relative to control animals, demonstrating the importance of antioxidant compounds in the treatment of Alzheimer’s disease [48]. In addition, autopsy studies of multiple sclerosis patients revealed that active lesions of the white matter and cerebral cortex, demyelination, and neurodegeneration were associated with the presence of oxidized lipids in myelin membranes and apoptotic oligodendrocytes [49]. Overall, oxidative stress and mitochondrial dysfunction have been extensively reported as major contributors to the neuronal loss observed in several neurodegenerative diseases [50,51,52,53,54,55,56,57,58,59].

#### 2.1.2. Neuroinflammation

Neuroinflammation is the complex innate immune response of neural tissue to foreign bodies of the body. This process plays a role in neural tissue fix and resolution. However, in neurological diseases, neuroinflammation becomes persistent and detrimental to neuronal cells [60].

The inflammatory process in the central nervous system (CNS) results primarily from the presence of chronically activated glial cells (astrocytes and microglia) in the brain. Glial cells are the most abundant and widely distributed cells in the CNS, which interact with neurons and immune cells, as well as with blood vessels. Microglia are immune cells of the brain, being the neural tissue’s defense system. Their main functions in the CNS include removal of accumulated or deteriorated neuronal and tissue elements, interacting with neurons, regulating synaptic processes, and maintaining brain homeostasis. Upon stimulation or alterations at the brain level, microglia are morphologically altered, and inflammatory molecules, cytokines, and chemokines are released, which leads to neuroinflammation [61].

Astrocytes play a direct and important role in mediating neuronal survival and function in neurodegenerative diseases. The function of astrocytes (neuroprotective or neurodegenerative functions) depends on the microenvironment that astrocytes and neurons share. Astrocytes release neurotrophic factors such as nerve growth factor (NGF), glial cell line-derived neurotrophic factor (GDNF), mesencephalic astrocyte-derived neurotrophic factor (MANF), neurotrophin-3, and basic fibroblast growth factor (bFGF), and metabolic substrates, such as lactate and glutathione, to counteract neuronal death. Additionally, they provide protection by siphoning off the excess of excitotoxic agents, such as glutamate, potassium, and calcium [62,63]. Nonetheless, when astrocytes undergo a state of gliosis in response to neuronal injury, they release cytokines and chemokines that are toxic to neurons, further contributing, together with microglia, to neuronal damage [63].

Neuroinflammation is present in many neurodegenerative diseases. In Alzheimer’s disease, increased levels of tumor necrosis factor (TNF)-α and lower levels TNF-β were detected in the cerebrospinal fluid (CSF) of mild cognitive impairment patients when compared with the controls [64]. Regarding Parkinson’s disease, *postmortem* analyses indicated that the levels of cytokines are significantly elevated in the *substantia nigra* of patients [65,66]. In amyotrophic lateral sclerosis patients, an increase in active microglia and astrocytes was observed [67,68]. Lastly, in the cuprizone-induce demyelination experimental autoimmune encephalomyelitis (EAE) model, activated microglia were found in lesions of the CNS and were associated with CNS inflammation in multiple sclerosis [69]. Overall, several studies reported the presence and contribution of neuroinflammation to the progression of distinct neurodegenerative diseases [70,71,72,73,74,75,76,77,78].

#### 2.1.3. Protein Misfolding

Protein misfolding and aggregation of specific proteins into toxic products is a common feature of neurodegenerative diseases. Under physiological conditions, cells are normally exposed to misfolded proteins (due to alterations in biogenesis, diseases-causing mutations, or endogenous inducers), but have the capability to counteract this effect by degrading or refolding misfolded proteins though the activity of chaperone proteins. However, under pathological stress, the protein misfolding promotes synaptic dysfunction and neuronal cell death. The mechanisms by which they exert their toxicity are not clearly defined, but it appears to act primarily by toxic gain-of-function and dominant-negative effects.

Depending on the type of protein involved and the pathology in question, its aggregation promotes different consequences. For example, in Alzheimer’s disease, Aβ peptide originating from the fragmentation of amyloid precursor proteins (APP) accumulates in the brain in the form of senile plaques [79]. As a consequence, the Aβ overproduction induces its aggregation into oligomers, forming amyloid plaques that are visible in pathologic samples [80]. These plaques are toxic and can induce inflammation, hyperphosphorylation of the tau protein, excitotoxicity, and oxidative stress, and in the presence of iron, can also promote ROS generation [80].

In Parkinson’s disease, α-synuclein is often found accumulated and aggregated and has several harmful effects. The phosphorylation and fibrilization of α-synuclein leads to Lewy bodies formation, which is mainly responsible for the death of dopaminergic neurons [81]. Additionally, α-synuclein can induce the loss of presynaptic proteins, the decrease of neurotransmitter release, the enlargement of synaptic vesicles, the inhibition of synaptic vesicle recycling, and also perturbations in calcium homeostasis [82]. Besides, it inhibits mitochondrial complex I, inducing the selective oxidation of ATP synthase and causing mitochondrial lipid peroxidation, leading to generation of ROS and cell death [83].

Several misfolded proteins are also associated with amyotrophic lateral sclerosis, such as SOD1, TDP-43, ubiquilin-2, and p62, which are produced through the unconventional repeat associated non-ATG translation of the repeat expansion in C9ORF72, which can promote the inhibition of essential cellular functions, leading to neuronal loss [84]. Mutations in SOD1 gene account for 20% of amyotrophic lateral sclerosis cases, and promote activation of caspases, cytoskeletal abnormalities, and mitochondrial dysfunction [85]. Although involving distinct proteins, protein misfolding was extensively reported as a pathophysiological mechanism present in distinct neurodegenerative diseases [86,87,88,89,90,91].

#### 2.1.4. Iron Overload and Ferroptosis

Iron is a metal widely distributed in biological systems and its high availability and chemical properties (capability to form complexes with organic ligands and favorable redox potential to switch between its ferrous and ferric states) makes it a key component in energy-generating processes. This metal plays a remarkably important role in cellular processes (such as neurotransmission, DNA synthesis, oxygen transport), apart from catalyzing many chemical reactions. Regulating iron levels by controlling its absorption, use, storage, and excretion is extremely important, as low or high levels of this metal can have harmful effects on the human body [92,93].

Iron has essential functions in the brain and, therefore, needs to cross the BBB to reach this organ. The most elucidating hypothesis of the passage of iron through the luminal membrane of the capillary endothelium is through the transferrin/transferrin receptor (Tf/TfR) pathway. This process starts with the binding of iron-Tf to the extracellular portion of TfR, followed by the endocytosis of the complex of iron-Tf-TfR, formation of endosome, and acidification of the microenvironment within endosome. Next occurs the dissociation of iron from Tf and the reduction of ferric iron (Fe^3+^) to Fe^2+^ by the ferrireductase six-transmembrane epithelial antigen of prostate 3 (STEAP3). Lastly, the translocation of Fe^2+^ across the endosomal membrane occurs, in a process mediated by the divalent metal transporter 1 (DMT1), forming the labile iron pool (LIP) that is located in cytoplasm. The excess intracellular iron is then stored in the form of ferritin and, when this metal is needed, it can be exported across the membrane via ferroportin (FPN) [94,95].

The regulation of iron levels is systemically controlled by hepcidin (which regulates its intestinal absorption), and cellularly by iron regulatory proteins (IRPs). These proteins bind to iron responsive elements (IREs) implicated in iron metabolism. When there is a decrease in iron levels, IRPs bind to the IRE located in the 5′ untranslated regions of the mRNA of iron-responsive proteins (such as FPN and ferritin), inhibiting the translation of these proteins, leading to a reduction in iron export and free iron storage. In contrast, IRPs bind to IRE in the 3′ untranslated regions of TfR1 and DMT1 mRNA, promoting the translation of TfR1 and DMT1, and consequently increasing the iron uptake [96]. A dysregulation of iron metabolism can lead to an imbalance in the normal iron redox status and levels of Fe^2+^, which can participate in the Fenton reaction, leading to a cycle between the two redox states and prompting the generation of •OH [92].

Several studies have demonstrated the involvement of iron (in excess) in the progression of neurodegenerative diseases. For example, Bao et al. observed a decrease in FPN expression in both brains of mouse model and Alzheimer’s disease patients, with concomitant iron deposition [97]. In Parkinson’s disease, Sofic et al. found that the levels of total iron and ferric iron were increased (176% and 225%, respectively) in the *substantia nigra pars compacta* of Parkinson’s disease patients, relative to age-matched controls [98]. Jeong et al. evaluated the accumulation of iron in SOD1G37R transgenic mice (representative of amyotrophic lateral sclerosis), and observed iron accumulation in the spinal cord of mice at 12 months of age. In addition, through a colorimetric ferrozine assay for the determination of the total iron amount, a 56% increase in iron levels was observed in SOD1G37R mice when compared to age-matched wild-type control animals [99]. Finally, using a cuprizone mouse model of multiple sclerosis, reduced immunofluorescence labelling for ferritin and reduced mRNA expression of ferritin heavy chain was reported in the animal’s *corpus callosumn* [100].

Recently, a new type of programmed cell death has been identified called ferroptosis. According to the Nomenclature Committee on Cell Death (NCCD), ferroptosis is “a form of regulated cell death initiated by oxidative perturbations of the intracellular microenvironment that is under constitutive control by GPX4 and which can be inhibited by iron chelators and lipophilic antioxidants” [101]. Iron and lipid peroxides are the main participants, but in a ferroptotic process, the depletion of glutathione, decrease in GPX4 activity, NADPH oxidation, and inhibition of System Xc- (an amino acid antiporter that exchanges extracellular L-cystine and intracellular L-glutamate across the plasma membrane, impacting the synthesis of glutathione) also occurs. Through System Xc- inhibition, the entry of cystine into cells is interrupted, decreasing its conversion to cysteine, which participates in the synthesis of glutathione, therefore, reducing the synthesis of this important antioxidant [102]. This type of cell death has been increasingly associated with neurodegeneration. A study performed by Ashraf et al. analyzed the occurrence of iron dyshomeostasis, augmented lipid peroxidation, and impaired System Xc- in Alzheimer’s disease patients. It was observed that the expression of iron-storage proteins was increased in Alzheimer’s disease patients when compared with the medial temporal cortex of cognitively normal samples, and the levels of 4-hydroxy-2-nonenal [4-HNE, a lipid peroxidation product) were also significantly increased. Nonetheless, the expression of DMT1 and FPN were decreased in Alzheimer’s disease patients, and an impairment of System Xc- was also observed [103]. In another study performed in zebrafish and in SH-SY5Y cells, 6-hydroxydopamine (6-OHDA, neurotoxin used to mimic PD) significantly reduced the levels of glutathione and increased the levels of iron and malondialdehyde (MDA, a lipid peroxidation marker), which indicates that this compound can induce ferroptosis in both models of Parkinson’s disease [104].

In order to understand the involvement of ferroptosis in amyotrophic lateral sclerosis, namely lipid peroxidation, a group measured 4-HNE levels in amyotrophic lateral sclerosis patients and observed an increased level of this lipid peroxidation product in the serum and cerebrospinal fluid of sporadic amyotrophic lateral sclerosis patients when compared with controls. In addition, the group observed that the levels of 4-HNE were elevated in advanced stages of the disease when compared with earlier or moderate disease stages, which means that the 4-HNE levels were positively correlated with the disease stage [105]. In the case of multiple sclerosis, dimethyl fumarate (an approved therapeutic for this disease) was reported to modulate ferroptosis [46]. For example, the administration of dimethyl fumarate (100 mg/kg/day, for 28 days) promoted a reduction in iron and MDA levels in the hippocampus of a rat model of chronic cerebral hypoperfusion, as well as increased glutathione and SOD levels. Besides, the decreased expression of System Xc-, GPX4, and FTH1 transporter observed in the hippocampus of the chronic cerebral hypoperfusion rat model was recovered following dimethyl fumarate treatment [46].

Alterations in iron homeostasis promote the pathophysiological effects observed in several neurodegenerative diseases [106,107,108]. Furthermore, the occurrence of ferroptosis as a type of recent cell death has been receiving increased attention given its apparent occurrence in neurodegenerative diseases and the positive effect of inhibitors of this process in the disease progression [109,110,111,112,113,114,115,116]. Targeting ferroptosis can thus be proposed as a potential new therapeutic target to stop/delay neurodegenerative disease progression.

## 3. Marine Derived Biomolecules with Antioxidant Properties

The origin of the inflammatory events that trigger several diseases, such as cancer, cardiovascular diseases, diabetes, and neurodegenerative diseases, is related to oxidative stress resulting from the high production of ROS and RNS, which are not counterbalanced by the body’s antioxidant defenses [117,118,119]. Thus, the understanding of oxidative stress mechanisms, as well as the discovery of new compounds with antioxidant properties, have been the focus of various investigations that have already demonstrated the existence of a strong relationship between the use of antioxidant compounds and the reduction of the risk of developing these diseases [117,120].

In recent years, the biotechnological industry has been searching for antioxidant compounds from natural sources to replace artificial antioxidants, such as butylated hydroxyanisole (BHA) and butylated hydroxytoluene (BHT), whose safety profiles are increasingly controversial as they have been associated with liver damage and carcinogenesis. In this context, natural antioxidant molecules extracted from marine bio-waste, in particular carotenoids, bioactive peptides, and polysaccharides, constitute promising alternatives to the synthetic antioxidants [5,117,118]. Table 1 presents examples of antioxidant biomolecules from marine organisms, and their properties and potential therapeutic applications.

### 3.1. Carotenoids

Carotenoids share a C40 isoprene structure, called a terpenoid, and are divided into carotenes, which consist only of hydrocarbons, and xanthophylls, which are oxygenated products of carotenes [4,7,117]. These lipophilic compounds of different colors (e.g., yellow, orange, and red) have been widely used in the pharmaceutical and biotech industries, mainly due to their antioxidant properties [7,121]. For instance, astaxanthin is a red xanthophyll predominantly isolated from the microalga *Haematococcus pluvialis*, which accumulates very high levels of this compound under stress conditions, such as high salinity, high temperature, and nitrogen deficiency. However, astaxanthin can also be extracted from marine bio-waste, including shrimps and crab shells, where it is responsible for their orange pigmentation [137,138]. Chemically, astaxanthin is a high lipophilic molecule with the IUPAC name 3,3′-dihydroxy-β-β-carotene-4,4′-dione, whose structure contains two rings with a hydroxyl group and a carbonyl group separated by an unsaturated chain of carbon–carbon double bonds. This specific configuration, namely polyene chain, confers to astaxanthin a powerful antioxidant activity in scavenging free radicals, being 40 and 100 times more effective as antioxidant than β-carotene and vitamin E, respectively [137,139,140,141,142]. For this reason, the use of astaxanthin has been highlighted in several investigations due to its valuable impact on human health, namely in the prevention of cancer and in reducing the risk of developing cardiovascular and neurodegenerative diseases [117,141,143].

β-carotene is the main carotenoid produced by the halotolerant microalgae *Dunaliella salina*, although it can also be found in turban shells [117,130]. This compound is recognized for its antioxidant activity, in particular, its great ability to eliminate ROS due to its structure with conjugated double bonds that allow accepting electrons of reactive species, transforming them into neutral species [117,144]. Several investigations have shown that, in addition to its antioxidant properties and potential in the prevention of neurodegenerative diseases, β-carotene has other benefits for human health, such as the prevention of liver fibrosis, acute and chronic coronary syndrome, and the protection of the skin against UV radiation [117,127,144].

### 3.2. Bioactive Peptides

Bioactive peptides are small proteins with various physiological functions, in particular antioxidant activity. Generally, these peptides contain 2 to 20 amino acid residues and have the ability to scavenge ROS, chelate metal ions, and inhibit lipid peroxidation [2,5,131].

In recent years, there has been much research focused on the use of bioactive peptides, obtained from the enzymatic hydrolysis of marine bio-waste, in the promotion of human health as well as the prevention of chronic diseases. In particular, collagen, a protein found in the structure of fish skin, bones, and scales, and its partially hydrolyzed form, gelatin, are rich in hydrophobic amino acids, which appear to have a high free radical scavenging capacity. Peptides derived from the gelatin of the skin of marine animals, such as flying squid (*Ommastrephes batramii*) and tuna (*Thunnus* spp.), have demonstrated high antioxidant activity, similar to that of the potent natural antioxidant α-tocopherol [2,131]. Collagen has gained great interest in the cosmetic industry, in anti-ageing creams, and in nutritional supplements for bone and cartilage regeneration, vascular and cardiac reconstruction, and skin substitutes [132].

### 3.3. Polysaccharides

Several studies have reported that polysaccharides derived from marine organism’s exhibit antioxidant activity, suggesting that these compounds could be used to mitigate diseases mediated by oxidative stress, such as liver damage, diabetes, obesity, colitis, some types of cancer, and neurodegenerative diseases [118].

Among the different polysaccharides that can be extracted from marine organisms, chitin is the most exploited as it can be easily obtained from the exoskeletons of marine arthropods, such as crustaceans, cuttlefish, and squid. Through chemical or enzymatic processes of chitin, it is possible to obtain its derivative chitosan, which is of interest to the pharmaceutical industry due to its anticancer, antimicrobial, anticoagulant, immunological, and antioxidant properties that enable it to act in the prevention of various diseases, including neurodegenerative ones [133,145].

## 4. Intranasal Lipid Nanoparticles Containing Marine Bioactive Compounds for the Management of Neurodegenerative Diseases

Marine organisms are considered a large reservoir of bioactive compounds with high therapeutic value, and several studies have demonstrated the efficacy of marine biomolecules with antioxidant properties in the prevention and treatment of different diseases. Some of these biomolecules have been described as having neuroprotective effects, and their use has been suggested for the prevention and treatment of neurodegenerative diseases [121,146,147]. Among these compounds, carotenoids, such as astaxanthin, fucoxanthin, and β-carotene, have gained particular interest due to their high antioxidant activity, which can prevent/delay the onset of oxidative stress-related diseases, such as neurodegenerative diseases [122,148].

Astaxanthin has a protective effect on neuronal cells, being able to prevent and modulate the severity of neuronal death following oxidative stress-induced injury related to a high level of ROS [121,122,123,149]. Furthermore, results from recent studies support the beneficial effect of astaxanthin on the activation of antioxidant mechanisms, increasing the levels or stimulating the activity of endogenous enzymes, such as SOD and CAT [122,124,150]. Recently, astaxanthin is receiving attention for its effect on the prevention or co-treatment of Alzheimer’s and Parkinson’s diseases. The administration of astaxanthin as an adjunctive therapy for Alzheimer’s dieses has demonstrated that the compound is able to attenuate microglial activation and simultaneously decrease the release of pro-inflammatory cytokines and reduce ROS levels. Similarly, the administration of astaxanthin as an adjuvant therapy for Parkinson’s disease suggested that the biological activity of this compound could neutralize the pathophysiological characteristics of the disease, revealing a promising therapeutic potential in preventing or delaying the onset of symptoms in patients with Parkinson’s disease [121,122,151].

Fucoxanthin and β-carotene have also been shown to have a protective effect on cells against oxidative stress due to their antioxidant activity that attenuates pro-inflammatory secretion by microglial cells and activates endogenous antioxidant enzyme mechanisms capable of inhibiting free radical-induced DNA oxidation [7,121,127,128,129].

Human studies on the beneficial effects of carotenoids in the treatment and prevention of neurodegenerative diseases showed that the use of an antioxidant supplement containing astaxanthin and β-carotene reduced ROS production and Aβ accumulation in Alzheimer’s disease patients, showing the potential of these compounds in the prevention and treatment of the disease [129,152,153,154].

In addition to carotenoids, chitosan extracted from marine bio-waste, whose hydrolysis results in the formation of chito-oligosaccharides (COS), has shown good neuroprotective properties, with anti-neuroinflammatory and anti-apoptosis effects, suggesting the potential of COS as protective agents against neurodegeneration [134,135,136,155].

### 4.1. Intranasal Administration

Despite the progress that has been made in investigations of the pathogenic mechanisms underlying neurodegenerative diseases, the development of effective molecules and/or delivery systems that stop or slow their progression remains limited. One of the main drawbacks associated with current treatments is the occurrence of adverse effects since high doses usually have to be administered for the molecules to reach the brain in therapeutically effective concentrations [156,157,158].

According to the Food and Drug Administration (FDA), more than 90% of new drugs used to treat CNS diseases have not been approved due to the difficulty of molecules to cross the BBB and reach the brain, especially hydrophilic, ionized, or high molecular weight ones [14,157,159,160,161,162].

For this reason, several studies have investigated alternative and effective strategies to improve drug transport to the CNS by avoiding passage through the BBB, such as using the intranasal route that allows direct passage from the nasal cavity to the brain [14,156,163,164]. In addition to this important benefit, this route has demonstrated other advantages, including easy and non-invasive administration, avoidance of gastrointestinal and hepatic metabolism, high drug bioavailability, large surface area available for drug absorption, and rapid onset of action. However, several factors may limit the use of this route, such as short residence time in the nasal cavity, the small volume available for administration, and enzymatic degradation [14,156,165,166,167]. The main advantages and limitations of the intranasal route are summarized in Table 2.

#### 4.1.1. Nose-to-Brain Transport

**Figure 2:**
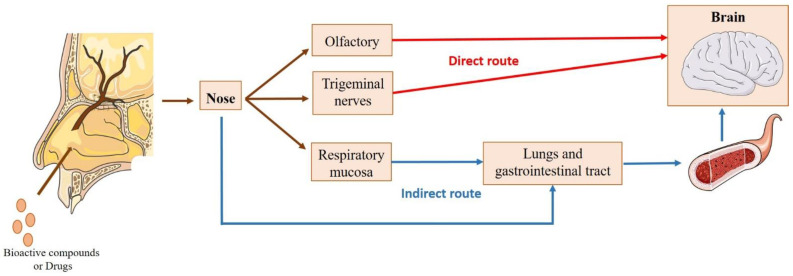
Possible transport routes of bioactive compounds or drugs to the brain after intranasal administration. Direct route: passage through the olfactory or trigeminal nerves, avoiding crossing the blood–brain barrier (BBB). Indirect route: absorption through the respiratory mucosa into the systemic circulation and across the BBB (adapted from Nguyen et al. [15,169]).

The mechanism of direct transport of compounds from the nose to the brain has been extensively studied, although there is no consensus about the exact path taken by the molecules upon intranasal administration (Figure 2). Several investigations have reported that, after entering the nasal cavity (in the vestibule region), the molecules undergo the mucociliary clearance mechanism. Subsequently, the molecules that are not eliminated in this process move to the posterior part of the cavity, where they contact the respiratory and olfactory regions. From here, they can be transported directly to the brain. Alternatively, molecules can be absorbed through the nasal mucosa into the bloodstream, having to cross the BBB to reach the brain [13,14,15,16,168].

The contribution of the indirect route to the transport of bioactive compounds or drugs to the brain is poor, since most molecules show difficulty in bypassing the BBB, especially hydrophilic and high molecular weight ones [16,157]. Thus, the direct route constitutes the main transport pathway to the brain. In particular, transport through the olfactory region, where the molecules pass through the olfactory nerves, has been described as the most relevant. The passage of the compounds through this pathway can be divided into two types of transport [13,14,15,16,170,171]: (i) intraneuronal, where olfactory neurons internalize the molecules by endocytosis or pinocytosis, releasing them by exocytosis and distributing them to the different brain regions; (ii) extraneuronal transport, where the molecules can cross the olfactory mucosa through the supporting cells (transcellular transport) or along the supporting cells (paracellular transport).

The passage of compounds through the trigeminal nerves (intracellularly or extracellularly) also constitutes a direct transport route to the brain, since this nerve has three different branches (mandibular, ophthalmic, and maxillary) that connect the nasal cavity to the CNS. However, this route is less significant for the transport of compounds to the brain [13,16,172].

#### 4.1.2. Factors Affecting Intranasal Absorption

Following intranasal administration, to ensure that direct transport of the compounds from the nose to the brain occurs, several factors must be considered, including the physicochemical properties of the molecules, the physiological and anatomical characteristics of the nasal cavity, and the particularities of the formulation [14].

Regarding the physicochemical properties of the molecules, factors such as molecular weight, lipophilic/hydrophilic characteristics, degree of ionization, and ability to solubilize in or penetrate mucus are important to determining the effectiveness of the nose-to-brain transport. In particular, molecules with a molecular weight greater than 1 kDa have difficulty in passing through the tight junctions between nasal cells, as opposed to molecules with a molecular weight of less than 300 Da, which pass easily through the nasal mucosa and are rapidly absorbed. The lipophilic/hydrophilic characteristics of the molecules, in particular those with a molecular weight between 300 Da and 1 kDa, determines the transport pathway these molecules follow, with lipophilic molecules passing through lipid-layered cells (transcellular pathway), and hydrophilic molecules passing cells through tight junctions (paracellular pathway) [13,16,171,173,174].

The physiological mechanism of mucociliary clearance of the nasal cavity is also one of the factors responsible for the inefficient transport of compounds to the brain since it can compromise the absorption of molecules in the nasal cavity [16,158,170]. Herein, the physicochemical properties of the molecules are quite decisive, since lipophilic molecules are less soluble in mucus, demonstrating a greater capacity for absorption in the nasal mucosa [166]. In addition, enzymes in the nasal cavity (carboxypeptidases and endopeptidases) promote the degradation of molecules, in particular peptides and proteins [16,174], while the expression of the efflux protein P-glycoprotein on the surface of ciliated nasal epithelium cells restricts absorption of the compounds [16,175]. Thus, when developing intranasal formulations, absorption promoters, enzyme inhibitors, and mucoadhesive agents can be used to improve the absorption of the compounds and increase their residence time in the nasal mucosa. Another approach that can be used is to encapsulate the compounds in lipid nanoparticles, which improves their absorption and protects them from enzymatic degradation [13,14,176]. Furthermore, intranasal formulations must have adequate viscosity and pH compatible with the nasal mucosa [6.4–6.8], avoiding irritation and discomfort after administration. They should also be isotonic so as not to interfere with normal cilia movement [16,177], composed of biocompatible and odorless excipients, and the administered volume should not exceed 200 µL [14,178].

Of note, intranasal formulations should be included in specific devices that direct them to the olfactory region of the nasal cavity, avoiding the losses that can occur after administration [14,168,179]. Nasal pharmaceutical dosage forms are generally presented in the form of drops and sprays. Drops, although simpler, show limitations in quantifying the amount of compound present in each drop, meaning that an excess can be easily administered. Thus, nasal sprays are preferable to drops because they are safer and easier to administer. However, the droplet diameter of the sprays should be greater than or equal to 10 µm to avoid deposition in the lower respiratory tract (i.e., in the lungs and bronchia) [16,180].

### 4.2. Using Lipid Nanoparticles for Nose-to-Brain Transport of Marine Bioactive Compounds

Several bioactive compounds and drugs proposed for the treatment of neurodegenerative diseases have limitations resulting from physicochemical instability and/or low bioavailability related to brain targeting difficulties [6,7,181]. To overcome these limitations, the use of lipid nanoparticles, namely SLN and NLC, have shown great efficiency in encapsulating and protecting these molecules, showing promising results in the treatment of these diseases. There have been several reviews published that provide detailed knowledge of the different characteristics and uses of lipid nanoparticle formulations. Interested readers are advised to read these works. Briefly, SLN contains a solid lipid matrix formed by a lipid, while NLC contain a solid lipid matrix formed by a solid and a liquid lipid, which allows to incorporate a larger amount of molecules and provides greater stability during storage when compared to SLN [13,14,17,170,182,183,184,185,186,187,188].

Although SLN and NLC share some advantages with other nanosystems, they have been showing better outcomes that are attributed to their particular characteristics. For example, they show superior biocompatibility than polymeric nanoparticles and inorganic nanoparticles; and they are more effective for brain targeting due to their lipidic nature that facilitates passage through the BBB. In addition, it has been reported that polymeric nanoparticles have less ability than SLN and NLC to prolong drug release, as the burst effect has been more frequently observed for the former. When compared to liposomes, the manufacture of SLN and NLC is cheaper as they use less expensive lipids. The latter also show greater long-term stability [12,13,14,15,16,17,185,189,190,191,192].

Several advantages have been described for the intranasal use of lipid nanoparticles, such as [16,158,166]: improved permeation through nasal mucosa; increased adhesion to the olfactory epithelium, avoiding mucocilliary clearance; protection of the encapsulated molecules from enzymatic degradation and P-glycoprotein efflux; ability to target the CNS, which increases the amount of compound reaching the brain, reducing the dose and frequency of administration. However, it is important that lipid nanoparticles have sizes below 200 nm and are composed of GRAS (generally recognized as safe) excipients in non-toxic concentrations so as not to damage the nasal mucosa [16,193]. The lipids and emulsifier(s) used must allow the formation of SLN or NLC with appropriate size, polydispersity index (PDI), and surface charge; high encapsulation ability and sustained release profile of encapsulated compounds, which is essential to the success of treatments [191]. Furthermore, after developing nasal lipid nanoparticles formulations, it is essential to assess their biocompatibility, first, in vitro, and then in vivo, to predict their clinical performance [14,194,195].

Several studies on the intranasal administration of natural bioactive compounds, obtained from different sources and encapsulated or on the surface of SLN and NLC, have demonstrated relevant outcomes in the treatment of neurodegenerative diseases. Specifically, for compounds obtained from marine bio-waste, only three studies were found (astaxanthin-loaded SLN, and SLN and NLC coated with chitosan), which shows the potential of this field. Table 3 summarizes the most relevant outcomes of these studies.

Although NLC have been preferred over SLN due to their apparent superiority for encapsulating compounds, the number of studies with these two types of nanoparticles is similar (Table 3). For instance, Bhatt et al. encapsulated astaxanthin in SLN for intranasal administration to improve brain targeting of the compound for the treatment of neurodegenerative disorders. The optimized astaxanthin-loaded SLN had a particle size of 213.23 nm and a PDI of 0.367. In vivo biodistribution studies, where the astaxanthin-loaded SLN were administered by the intravenous and intranasal routes, indicated that 1 h after administration, a higher concentration of astaxanthin was achieved in the brain with the intranasal formulation (1.70 ± 0.1312% injected dose/gram organ), compared to the intravenous (0.844 ± 0.12 injected dose/gram organ). These results demonstrated that intranasal administration of astaxanthin-loaded SLN improved the brain uptake of astaxanthin compared to intravenous administration, suggesting that direct nose-to-brain transport occurs. Furthermore, in vitro studies in pheochromocytoma-12 cell line (PC12) demonstrated the antioxidant potential of astaxanthin-loaded SLN against H_2_O_2_ induced toxicity. In conclusion, the results of these investigations support the use of astaxanthin-loaded SLN for brain targeting, which allows protection against various neurodegenerative diseases [141]. In another study, Sun et al. developed an in situ gel with paeonol-loaded SLN for direct nose-to-brain transport. Paenol is a phenolic compound with therapeutic potential in different neurodegenerative diseases. The nanoparticles developed had a particle size of 166.79 ± 2.92 nm and a PDI of 0.241 ± 0.030. In vitro studies showed that in situ gel with PAE-loaded SLN exerted low toxicity in RPMI 2650 cells. In vivo biodistribution studies showed that the effective accumulation of the in situ gel in the brain area after intranasal administration proved that it could effectively transport the paenol-loaded SLN to the brain, suggesting its potential use in the treatment of neurodegenerative diseases [198].

Regarding Parkinson’s disease, Trapani et al. studied the effects of co-administration of dopamine combined with antioxidant grape seed-derived polyphenol compounds (GSE) encapsulated in SLN for intranasal administration as a novel approach in the treatment of this disease. The developed dopamine/GSE-loaded SLN had a particle size of 184 ± 34 nm and a PDI of 0.32 ± 0.07, and showed no toxicity in olfactory ensheathing cells (OECs) and neuroblastoma (SH-SY5Y) cells. Furthermore, in vitro evaluation of the effects on cell viability of incubating dopamine/GSE-loaded SLN and the oxidative stress-inducing neurotoxin 6-hydroxydopamine (6-OHDA) (100 µM) clearly demonstrated that DA/GSE-loaded SLN increased cell viability compared to cells treated with 6-OHDA alone. Therefore, it was concluded that dopamine/GSE-loaded SLN are promising for direct nose-to-brain transport of the tested compounds in the treatment of Parkinson’s disease [196]. In another study, Junior et al. combined the anti-inflammatory properties of geraniol (GER), a natural compound known to promote the survival of dopaminergic neurons, with the mitochondrial rescue effects of ursodeoxycholic acid (UDCA) to improve the treatment of Parkinson’s patients. The nanoparticles developed GER/UDCA-loaded SLN had a particle size of 121 ± 8.4 nm and a PDI of 0.164 ± 0.03. In vivo studies with intranasally administered of these nanoparticles demonstrated selective uptake of GER/UDCA into the cerebrospinal fluid, suggesting that direct nose-to-brain transport of the compounds occurs. Furthermore, histopathological evaluation demonstrated that, in contrast to pure GER, nasal administration of GER/UDCA-loaded SLN did not damage the structure of the nasal mucosa. In conclusion, these studies indicate that co-encapsulation of GER/UDCA in SLN may constitute an effective non-invasive approach to direct the compounds to the brain in the treatment of Parkinson’s disease [199].

Concerning Alzheimer’s disease, Saini et al. developed ferulic acid-loaded SLN coated with chitosan to improve the efficacy of this natural compound in the management of Alzheimer’s disease. The optimized ferulic acid/chitosan-loaded SLN had a particle size of 184.9 nm. In vivo pharmacodynamic studies showed a marked improvement in cognition after administration of ferulic acid/chitosan-loaded SLN compared to uncoated ferulic acid-loaded SLN and pure ferulic acid solution. In addition, administration of ferulic acid intranasally was found to be more beneficial in upregulating biochemical parameters over the oral route and resulted in higher brain concentrations of the compound compared to uncoated ferulic acid-loaded SLN. Thus, surface coating the SLN with chitosan originated remarkably higher brain levels of ferulic acid, probably owing to a prolonged retention time of the formulation in the nasal cavity, which is due to the SLN positive charge provided by the chitosan coating [197].

Malvajerd et al. encapsulated curcumin in SLN and NLC to increase the concentration of compound in the brain due to its great therapeutic potential to manage CNS diseases. The developed curcumin-loaded SLN and NLC had particle size and PDI of 204.76 ± 0.36 nm and 0.194 ± 0.04 for curcumin-loaded SLN, and 117.36 ± 1.36 nm and 0.188 ± 0.020 for curcumin-loaded NLC, respectively. The in vitro toxicity of the formulations on rat fetal fibroblast cells was evaluated, and high cell viability was observed for concentrations up to 10 μg/mL. Furthermore, in vivo studies showed that curcumin-loaded NLC were able to increase brain uptake of the compound more than 4-fold compared to curcumin-loaded SLN. In view of these results, it was concluded that the use of curcumin-loaded NLC in the treatment of CNS diseases is promising [200]. In another study, Abourehab et al. optimized nicergoline-loaded sesame oil-based NLC for intranasal administration to achieve synergistic and enhanced neuroprotective properties, since nicergoline is described to be used in the treatment of dementia and other cerebrovascular diseases and sesame oil slows and reverses the cognitive symptoms of neurodegenerative diseases. The nicergoline-loaded NLC had a particle size of 111.18 ± 6.33 nm and a PDI of 0.251 ± 0.04. In vivo bioavailability and brain distribution studies showed a 4.57-fold increase of the compound in the brain compared to a nicergoline-free solution, after intranasal administration of the formulation to rats. The results of the in vivo experiments also showed effective brain targeting efficiency (BTE) and direct transport percentage (DTP) of 187.3% and 56.6%, respectively, indicating the efficacy of the nicergoline-loaded NLC for direct nose-to-brain transport [201].

Recently, El-Enin et al. optimized berberine-loaded NLC coated with chitosan for brain targeting via the intranasal route, as recent investigations have shown this natural compound to be effective against Alzheimer’s disease, among other neurodegenerative diseases. The developed berberin/chitosan-loaded NLC had a particle size of 180.9 ± 4.3 nm. In vivo brain accumulation experiments showed that animals treated intranasally with berberin/chitosan-loaded NLC had substantially higher levels of the compound in the brain compared to those that were administered intranasally with a berberine solution. According to these results, the researchers concluded that berberin/chitosan-loaded NLC might be a successful approach to potentiate the effect of intranasal berberin in the treatment of CNS diseases, such as Alzheimer’s [202].

## 5. Conclusions

The use of marine bio-waste with antioxidant properties promotes greater sustainability and awareness of the importance of recovery and valorization of waste resulting from the processing of marine organisms and, in particular, the concept of circular economy.

Intranasal administration of lipid nanoparticles, namely SLN and NLC, containing natural bioactive compounds obtained from different sources has potential in the prevention and treatment of neurodegenerative diseases, as these compounds can be transported directly from the nose to the brain, without crossing the BBB. In particular, for bioactive compounds obtained from marine bio-waste, few studies have been reported, showing the open potential of this research area. More in-depth knowledge about the potential neuroprotective effects of bioactive compounds from marine bio-waste is needed to enable their future clinical use.

Clinical studies are needed to evaluate the efficacy of using bioactive compounds loaded in SLN or NLC for intranasal administration. Although preclinical studies in animals have already shown evidence of the occurrence of a direct transport of molecules from the nose to the brain, the exact mechanism of this transport is not fully understood and its efficacy in humans remains undefined. Further knowledge should be gained about the effects of these nanoparticles within the body, including the degradation/elimination of excipients, release of molecules, and interactions with organs and tissues. It is also important to highlight the fact that anatomical and physiological differences between animals and humans can provide incomplete information that may lead to the failure of clinical trials.

Noteworthy, although not excluding the need to perform in vivo studies, investigations conducted in 3D models of the human nasal cavity may provide a deeper understanding of the factors that interfere with intranasal administration, such as, for example, the type and angle of the administration device, and the inclusion of mucoadhesive excipients in the formulations.

Despite the lacks identified, in the near future, the use of SLN and NLC via the nose-to-brain route could play a pivotal role in improving treatments of neurodegenerative diseases.

## Figures and Tables

**Table 1:** Examples of antioxidant biomolecules from marine organisms.

Biomolecule	Natural Source	Therapeutic Properties and Potential	References
Astaxanthin	Shrimp/crab shells *Haematococcus pluvialis*	Antioxidant and anti-inflammatory properties. Prevention and treatment of cardiovascular and neurodegenerative diseases.	[117,121,122,123,124,125]
Fucoxanthin	Brown algae *Laminaria japonica*	Antioxidant and anti-inflammatory properties. Prevention and treatment of neurodegenerative diseases.	[7,121,126]
β-carotene	Turban shell Microalga *Dunaliella salina*	Antioxidant properties. Prevention of liver fibrosis, acute and chronic coronary syndrome, and neurodegenerative diseases. Protection against UV radiation.	[117,127,128,129,130]
Collagen	Cod skin	Antioxidant properties. Anti-aging.	[2,131,132]
Gelatin	Tuna (*Thunnus* spp.) Flying squid (*Ommastrephes batramii*)	Antioxidant and anti-proliferative properties. Prevention of cancer.	[131]
Chitin	Crustaceans Cuttlefish Squid pen	Antioxidant, anticancer, antimicrobial, and anticoagulant properties. Immune system boosting. Wound healing.	[133,134,135,136]

**Table 2:** Main advantages and limitations of the intranasal route.

Advantages	Limitations
Non-invasive and easy self-administration; Possibility of transporting drugs directly to the CNS, avoiding the need to cross the BBB; Prevention of hepatic first-pass metabolism of drugs; Avoidance of degradation of drugs in the gastrointestinal tract; Fast drug absorption; High bioavailability of the drugs, providing the administration of low doses.	Small volume administration (˂200 μL); Rapid elimination of drugs due to the mucociliary clearance mechanism; Enzymatic degradation of drugs by P-glycoprotein, carboxypeptidases or endopeptidases; Low permeability for drugs with high molecular weight (>1 kDa); Interindividual variability.

BBB: blood–brain barrier; CNS: central nervous system.

**Table 3:** Examples of the most relevant results from studies with natural bioactive compounds, encapsulated or on the surface of intranasal lipid nanoparticles (SLN or NLC), for the treatment of neurodegenerative diseases.

Type of Lipid Nanoparticle	Natural Bioactive Compound	Relevant Outcomes	Reference
SLN	Astaxanthin	In vitro studies demonstrated the antioxidant potential of astaxanthin-loaded SLN against H_2_O_2_ induced toxicity. In vivo biodistribution studies demonstrated a higher accumulation of astaxanthin-loaded SLN in the brain after intranasal administration (1.70 ± 0.13% injected dose/gram organ), when compared to the intravenous route (0.844 ± 0.12% injected dose/gram organ).	[141]
SLN	Dopamine combined with antioxidant grape seed-derived polyphenol compounds (GSE)	In vitro studies demonstrated that the dopamine/GSE-loaded SLN formulations did not exert toxicity on olfactory ensheathing cells (OECs) and on neuroblastoma cells (SH-SY5Y). Co-administration of dopamine/GSE-SLN and the oxidative stress-inducing neurotoxin 6-hydroxydopamine (6-OHDA) (100 µM) clearly demonstrated that formulation of dopamine/GSE-SLN determined an increase in cell viability, compared to cells treated with 6-OHDA alone.	[196]
SLN coated with chitosan	Ferulic acid	In vivo, the ferulic acid intake via the intranasal route was found to be much more beneficial in upregulating the biochemical parameters, in relation to the oral treatment. Intranasal ferulic acid/chitosan-loaded SLN showed superior concentration of ferulic acid in the rat’s brain, when compared to the uncoated ferulic acid-loaded SLN.	[197]
SLN in situ gel	Paeonol	In vitro studies with paenol-loaded SLN and an in situ gel with paenol-loaded SLN showed a low level of toxicity in RPMI 2650 cells. In vivo biodistribution studies showed an effective accumulation of the in situ gel in the brain, after intranasal administration.	[198]
SLN	Geraniol combined with ursodeoxycholic acid (GER/UDCA)	In vivo studies demonstrated a selective uptake of GER/UDCA to the cerebrospinal fluid, after nasal administration of GER/UDCA-loaded SLN.	[199]
SLN and NLC	Curcumin	In vitro studies with curcumin-loaded SLN and NLC showed no toxicity in mouse fetal fibroblast cells for concentrations up to 10 µg/mL. In vivo studies showed that curcumin-loaded NLC were able to promote the brain uptake of curcumin more than 4-fold, compared to curcumin-loaded SLN.	[200]
NLC	Nicergoline	In vivo, bioavailability and brain distribution studies of nicergoline-loaded NLC showed a 4.57-fold increase of the compound in the brain, compared to nicergoline solution. Results of in vivo studies indicated efficient direct nose-to-brain transport, with brain-targeting efficiency (BTE) and direct transport percentage (DTP) of 187.3% and 56.6%, respectively.	[201]
NLC coated with chitosan	Berberine	In vivo studies showed that animals treated with intranasal berberine/chitosan-loaded NLC had substantially higher levels of the compound in the brain, compared to animals treated with intranasal berberine solution.	[202]

## Data Availability

Not applicable.
